# A one-step, one-tube real-time RT-PCR based assay with an automated analysis for detection of SARS-CoV-2

**DOI:** 10.1016/j.heliyon.2020.e04405

**Published:** 2020-07-07

**Authors:** Bhasker Dharavath, Neelima Yadav, Sanket Desai, Roma Sunder, Rohit Mishra, Madhura Ketkar, Prasanna Bhanshe, Anurodh Gupta, Archana Kumari Redhu, Nikhil Patkar, Shilpee Dutt, Sudeep Gupta, Amit Dutt

**Affiliations:** a Integrated Cancer Genomics Laboratory, Advanced Centre for Treatment, Research, and Education in Cancer, Kharghar, Navi Mumbai, Maharashtra, 410210, India; b Shilpee Dutt Laboratory, Advanced Centre for Treatment, Research, and Education in Cancer, Kharghar, Navi Mumbai, Maharashtra, 410210, India; c Haematopathology Laboratory, Advanced Centre for Treatment, Research, and Education in Cancer, Kharghar, Navi Mumbai, Maharashtra, 410210, India; d Department of Medical Oncology, Advanced Centre for Treatment, Research, and Education in Cancer, Kharghar, Navi Mumbai, Maharashtra, 410210, India; e Homi Bhabha National Institute, Training School Complex, Anushakti Nagar, Mumbai, Maharashtra, 400094, India; f Adjunct Faculty, Institute of Advanced Virology, Kerala State Council for Science, Technology and Environment, Govt of Kerala, Thonnakkal, Kerala, 695317, India

**Keywords:** Infectious disease, Microbial genomics, Genomics, Molecular biology, Virology, Diagnostics, SARS-CoV-2, Real-time RT- PCR, COVID-19 detection assay, COVID qPCR analyzer, Graphical user interface (GUI)

## Abstract

Early diagnosis of SARS-CoV-2 infected patients is essential to control the dynamics of the COVID-19 pandemic. We develop a rapid and accurate one-step multiplex TaqMan probe-based real-time RT-PCR assay, along with a computational tool to systematically analyse the data. Our assay could detect to a limit of 15 copies of SARS-CoV-2 transcripts—based on experiments performed by spiking total human RNA with *in vitro* synthesized viral transcripts. The assay was evaluated by performing 184 validations for the SARS-CoV-2 *Nucleocapsid* gene and human *RNase P* as an internal control reference gene with dilutions ranging from 1-100 ng for human RNA on a cohort of 26 clinical samples. 5 of 26 patients were confirmed to be infected with SARS-CoV-2, while 21 tested negative, consistent with the standards. The accuracy of the assay was found to be 100% sensitive and 100% specific based on the 26 clinical samples that need to be further verified using a large number of clinical samples. In summary, we present a rapid, easy to implement real-time PCR based assay with automated analysis using a novel COVID qPCR Analyzer tool with graphical user interface (GUI) to analyze the raw qRT-PCR data in an unbiased manner at a cost of under $3 per reaction and turnaround time of less than 2h, to enable in-house SARS-CoV-2 testing across laboratories.

## 1 Introduction

Diagnostics can play an important role in the containment of COVID-19, enabling rapid implementation of control measures that limit the spread through case identification, isolation, and contact tracing (*i*.*e*., identifying people that may have come in contact with an infected patient). The COVID-19 causative agent is a novel Coronavirus belonging to genus beta-coronavirus of *Coronaviridae* family having shown genome sequence similarities of about 80%, 50% and ~90% with the previously reported SARS-CoV, MERS-CoV and SARS-like bat-CoV respectively [1, 2, 3, 4]. The novel coronavirus, termed as SARS-CoV-2 by the International Committee on Taxonomy of Viruses (ICTV) has created havoc around the globe causing at least 347,011 deaths out of 5,526,224 confirmed cases across 215 countries as of 26^th^ May 2020 [5].

The symptoms expressed by COVID-19 patients- fever (89%), cough (68%), fatigue (38%), sputum production (34%), and shortness of breath (19%) --are nonspecific and cannot be used for an accurate diagnosis [6]. Molecular techniques, on the other hand, are more suitable for accurate diagnoses because they can target and identify specific pathogens. The most commonly used targets for primer/probe development in case of SARS-CoV-2 are from conserved viral genome including *ORF1ab* *gene*, *RdRP* gene (RNA dependent RNA polymerase), *N* gene (Nucleopcapsid) and *E* gene (Envelope) [2, 7, 8]. Apart from conventional RT-PCR and quantitative RT-PCR, various studies have reported modifications like nested rRT-PCR, RT-PCR with locked nucleic acid probes and RT-Loop Mediated isothermal Amplification (RT-LAMP) for detection of Coronaviruses [9, 10, 11], e.g. the RT-LAMP method for detection of MERS-CoV with sensitivity as high as ~3.4 copies of RNA/reaction, a microarray-based methodology to detect strains of coronaviruses simultaneously, CRISPR effector Cas13 based SHERLOCK for viral RNA detection with isothermal amplification, and more recently for the COVID-19 [9, 12, 13, 14]. Despite such varied efforts to increase sensitivity and specificity of the viral diagnostics, there is still an unmet need for user-friendly and cost-effective detection method to combat the ongoing catastrophe created by SARS-CoV-2.

Analyzing the human coronavirus genome sequences, we have designed a combination detection method involving a novel primer targeting SARS-CoV-2 *Nucleocapsid N* gene sequence with two primer pairs each probing to another non-overlapping region of SARS-CoV-2 *Nucleocapsid N* gene and *ORF1ab* gene respectively. Using primers targeting SARS-CoV-2 specific regions, we present a comparative account of a DNA-binding dye SYBR Green I and sequence-specific dually fluorophore-labelled hydrolysis (TaqMan) probe-based qRT-PCR to help develop a one-step, one tube multiplex real-time RT-PCR reaction based assay for simple, reliable and rapid detection of the COVID-19 virus. For quantitative assessment of the data generated from the RT-PCR assay, we developed COVID qPCR Analyzer, a graphical user interface (GUI) based tool for reproducible downstream analysis and automated report generation of the analysed samples.

## 2 Materials and methods

### 2.1 Patient samples

Validation of the one-step, one-tube, multiplex probe-based real-time PCR assay presented in this study was performed on total RNA derived from swabs obtained from 26 patients who were referred for COVID-19 testing at the Department of Haematopathology Laboratory, ACTREC-TMC, as a routine service, which is a Government approved and authorized testing laboratory for COVID-19 by the Indian Council of Medical Research. This was part of standard care; therefore, no IRB approval was obtained. All patient samples obtained were anonymized and no patient specific characteristics including the age, gender, or any clinico- pathological features of the patients were accessed.

### 2.2 SARS-CoV-2 specific sequence selection, primer and probe design

Genome sequences of 93 SARS-CoV-2, 6 human-specific SARS viruses and 27,399 related SARS/SARS-like viruses were downloaded from NCBI-Virus database [15]. These included the 56 reference genomes of the *Coronavirdae* family of which the coding regions were also separately downloaded for analysis. Multiple sequence alignment was performed using the MAFFT tool [16] to identify conserved regions among the genes and Nucleotide BLAST was used for sequence comparisons. Three target regions for detection were selected using the Indian isolate of the SARS-CoV-2 genome (GenBank reference: 29854). Primers were designed using the Primer3plus [17] for the *Nucleocapsid* gene region (N1). The other two regions from the *Nucleocapsid* gene and *ORF1ab* gene (N2, N3) respectively were selected from literature (Supplementary Table S1) [18]. Dual-labelled TaqMan probes (Eurofins Scientific India Pvt Ltd, Bangalore) were designed for N1, N2 and C1 (Supplementary Table S2) using PrimerQuest tool (https://eu.idtdna.com/pages/tools/primerquest). *In silico* validation was performed by subjecting the templates generated from the selected regions/primer pairs to local blast (Nucleotide BLAST algorithm) based alignment against the available complete SARS-CoV-2 genome isolates and other SARS viruses having a human and non-human host.

### 2.3 Cloning and viral template synthesis

SARS-CoV-2 positive template control for SARS-CoV-2 *Nucleocapsid* gene (N1 and N2) and *ORF1ab* gene (N3) were generated in two-steps. The single-stranded synthetic DNA fragments (Supplementary Table S3) were concatenated and amplified by overlap extension PCR (OE-PCR) [19] for cloning in pJET.2/blunt cloning vector (CloneJET PCR Cloning Kit, Cat. K1232) as per manufacturer's instruction. N1, N2 and N3 synthetic fragment cloned plasmids were linearized at 3′ end using XbaI enzyme (NEB, Cat. R0145S). *In Vitro* Transcription (IVT) reaction was set up using T7 RNA polymerase (Promega, Cat. 207B) with each gel-purified plasmid and incubated at 37 °C for 2 h as described previously [20]. The resultant mixture was subjected to DNase I treatment (rDNase I DNA-free kit, Cat. No. 2224G, Invitrogen BioServices India Pvt. Ltd.) for digestion of left-over plasmid DNA and purification was confirmed by denaturing RNA agarose gel electrophoresis.

### 2.4 cDNA synthesis and qRT-PCR assay

For each IVT synthesized N1, N2 and N3 transcripts, cDNA was synthesized using PrimeScript cDNA synthesis kit (Cat. RR037A, Takara) with range of concentrations of IVT RNA (1pg, 10pg, 100pg and 1000pg) and total human RNA (50ng) as per manufacturer's protocol. qPCR with each synthetic viral DNA/IVT RNA fragment with or without either human cDNA or genomic DNA (10ng) as template was performed using SYBR Green Master Mix (Kapa SYBR Fast Universal qPCR kit, Cat. KK4601). *RNase P* (C1) was used as an internal reference control. Each 20ul reaction contained 10μl 2X SYBR green master mix (Kapa SYBR Fast Universal qPCR kit, Cat No. KK4601), 1μl of forward and reverse primers each along with either 1μl of human cDNA/gDNA, 1μl of synthetic viral DNA template and 6μl of nuclease-free water or 8μl of cDNA prepared from viral IVT RNA in the reaction mixture.

### 2.5 One-step qRT-PCR assay

Dual labelled TaqMan probes with 5′-6-FAM fluorescent dye and 3′- BHQ-1 quencher for two SARS-CoV-2 target sequences N1 and N2 were used for the detection of viral RNA. For internal reference control, a pair of primers and TaqMan probe for *RNase P* (C1), labelled with 5′-HEX fluorescent dye and 3′-BHQ-1 quencher were used. Quantitative RT-PCR was performed with a range of concentrations of SARS-CoV-2 synthetic DNA/RNA/both (1pg, 10pg, 100pg and 1000pg) along with human genomic DNA (10ng)/total RNA (50ng)/both as template using KiCqStart One-Step Probe RT-qPCR ReadyMix (Cat. KCQS07, Merck Life Science Private Limited). Fluorescent dye program was selected as 6-FAM for TaqMan probes, N1 and N2, and VIC was selected for TaqMan probe, C1. For both the probes, no quencher option was selected in the program.

### 2.6 One-step, One-tube Multiplex qRT-PCR assay

For multiplexing qRT-PCR with TaqMan probes, each reaction mixture was prepared with a range of concentrations (1 pg, 10 pg, 100 pg and 1000 pg) for SARS-CoV-2 synthetic DNA/IVT RNA. Thermocycler conditions and fluorescence dye settings were selected similar to One-step TaqMan RT-PCR in QuantStudio 5 Real-Time PCR system (Applied BioSystems, Cat no. A34322). In addition to the test samples, a positive control, negative control and no template control well make a set of reference sample for each assay. The positive control reference includes 50 ng total human RNA spiked with *in vitro* transcribed N1 and N2 transcripts; the negative reference includes 50 ng total human RNA; and the no-template control consists of water instead of human RNA.

### 2.7 Development of COVID qPCR analyzer tool

The tool is designed to process the qRT-PCR raw data (XLS format) to generate a report stating the positivity of the sample. The Ct values for individual wells on the plate (assigned to specific samples) are extracted for the viral test and control probe. Firstly, the tool checks if the Ct value of positive control well, is less than the threshold for the test probe and has undetermined Ct values for both test and control probe for the specified no template control well. In brief, the following algorithm is used to call a sample as COVID-19 positive/negative or to assess if the validity of the PCR run; a) a sample is “positive” in case the test gene probe is less than the Ct threshold (irrespective of control probe value), b) a sample is called “negative”, if Ct value for test greater than a threshold and of that of control probe is less than the Ct threshold set. In case the Ct value for the test and control probe is undetermined or greater than the threshold, the run for the sample is considered “invalid”. Implementation of the tool, including the graphical user interface has been done using R programming (www.r-project.org/) and gWidgets2 (https://github.com/jverzani/gWidgets2). The basic script to parse and extract data from qRT-PCR raw data is adopted from the project (https://github.com/TaylorMatte/Quant6-Covid_Analysis), of a recently published study [21]. The COVID qPCR Analyzer tool (instruction manual provided as Supplementary File1) along with the test data and output files is freely available for download at www.actrec.gov.in/pi-webpages/AmitDutt/Covid/Analyzer.html.

## 3 Results

### 3.1 Generation of SARS-CoV-2 synthetic viral template

**Figure 1:**
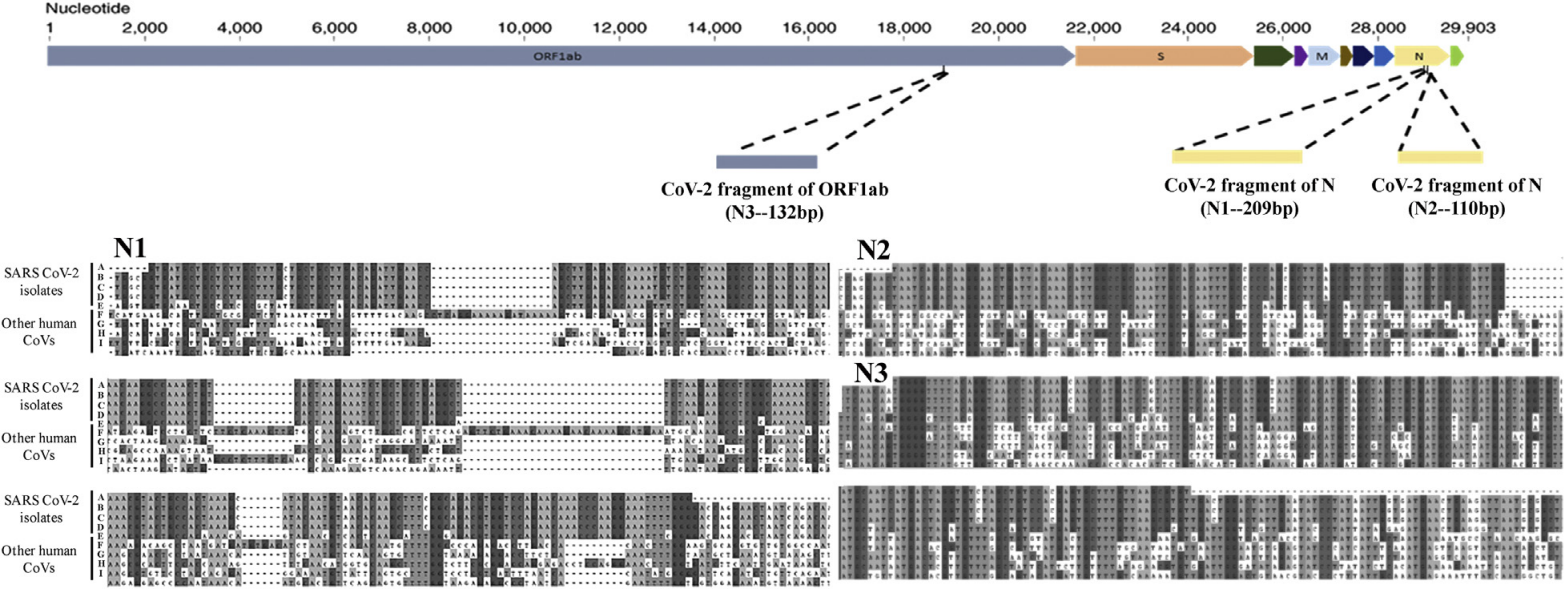
Sequence based analysis to identify the conserved regions for specific detection of SARS-CoV-2. Representation of the SARS-CoV-2 genome to the scale (adapted from [32]). The marked locations for N1-209 bp, N2-110 bp and N3-132 bp in the SARS-CoV-2 genome are approximated with respect to the genomic co-ordinates. The maps below represent region from the multiple sequence alignment (MSA) of N1 and N2 target templates with the *Nucleocapsid* (*N*) gene and N3 target template with the *ORF1ab* gene, respectively. MSA is performed between the genes from SARS-CoV-2 reference genome NC_045512 (A), isolates of SARS-CoV-2 from Indian samples https://www.ncbi.nlm.nih.gov/nuccore/MT0504932, https://www.ncbi.nlm.nih.gov/nuccore/MT012098 (B,C); SARS-CoV 2002–2004 NC_004718 (D); MERS NC_019843 (E); human coronavirus HKU1 NC_006577 (F); human coronavirus OC43 NC_006213 (G); human coronavirus 229E NC_002645 (H); human coronavirus NL63 NC_005831 (I); and the respective target regions.

Comparative sequence analysis was performed to understand the evolutionary conservation within the SARS-CoV-2 genomes and divergence among the genomes across the various members of *Coronaviridae* family and other human infecting SARS viruses. We selected a region of the SARS-CoV-2 *Nucleocapsid* gene, which was conserved across all the available CoV-2 isolate genomes (RefSeq ID NC_045512.2: 28917-29134). We additionally selected two regions reported earlier in the literature [18]. Multiple sequence alignment between the three templates (hereafter called N1, N2 and N3) and respective genic regions in all SARS-CoV-2 isolates and other human infecting SARS viruses has been shown in Figure 1. Basic criteria for selection of these three regions was conservation across all the available SARS-CoV-2 genomes and non-conservation between other human SARS viruses. Primers for Overlap extension PCR and 3 specific primer pairs for real time PCR were designed for N1, N2 and N3 regions to amplify unique N1 (209bp), N2 (110bp) and N3 (132bp) fragments as SARS-CoV-2 synthetic DNA representing the *Nucleocapsid* gene and *ORF1ab* gene respectively (Figure 1). Overlap extension PCR with Q5 DNA polymerase was used to amplify each of these concatenated oligonucleotide fragments. The amplified products were cloned into pJET1.2/Blunt vector and confirmed on an agarose gel after restriction digestion with a combination of enzymes PstI, HindIII and XbaI. The viral synthetic DNA templates were transcribed *in vitro* using T7 DNA dependent RNA polymerase after linearizing the plasmid vector with XbaI. RNA agarose gel electrophoresis for the IVT RNAs confirmed the sizes and purity of transcripts in both DNase I treated and untreated samples (Supplementary Figure S1).

### 3.2 SARS-CoV-2 detection by quantitative real-time PCR

**Figure 2:**
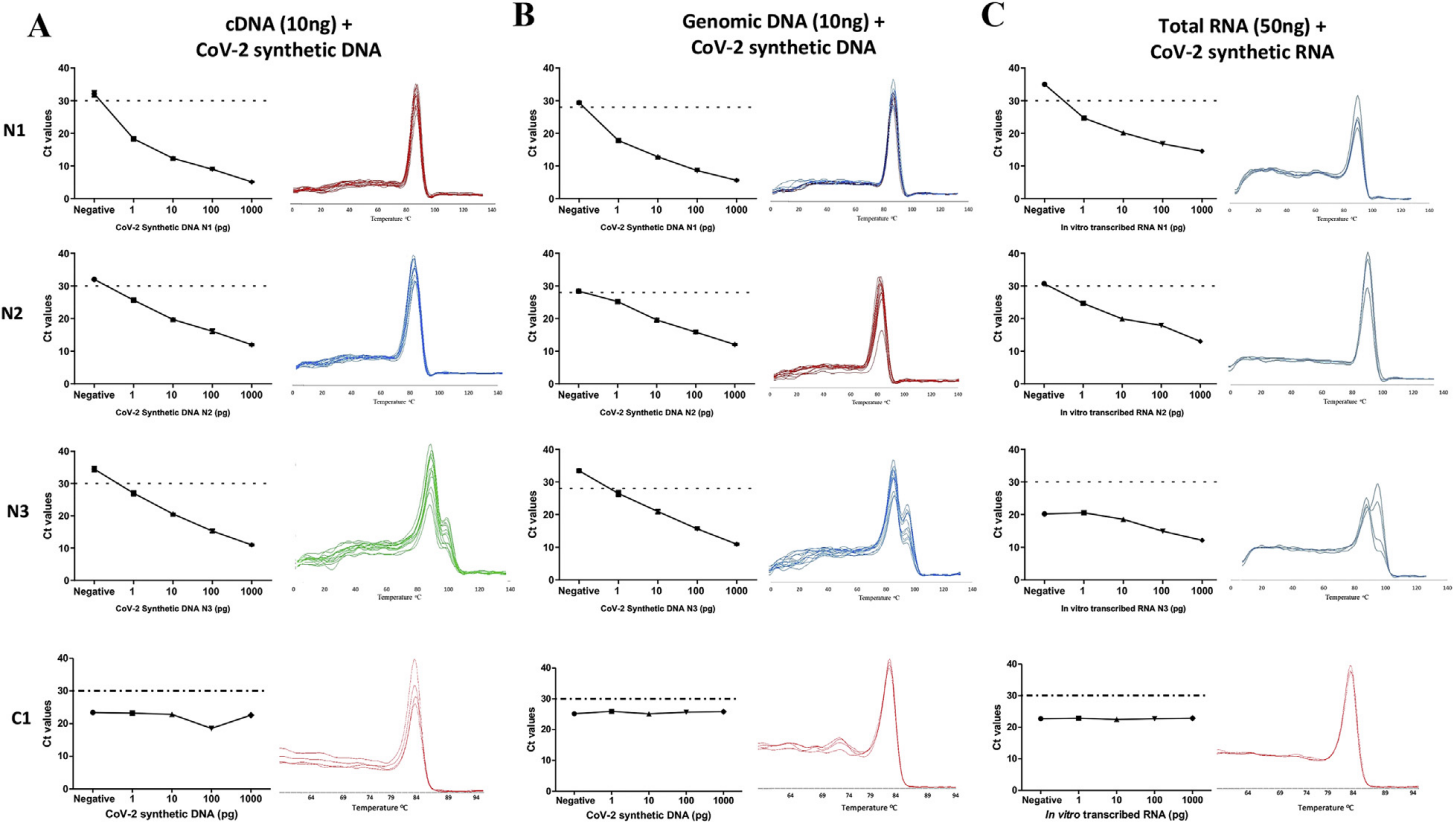
qRT-PCR using SYBR Green I dye based detection approach. Real time PCR to determine the sensitivity and specificity of SARS-CoV-2 detection primers of N1, N2, N3 and C1 (*RNase P* internal control) using SYBR Green I dye. Series of dilutions of each synthetic DNA fragment (1pg, 10pg, 100pg and 1000pg) were used in the background of (a) MCF-7 cell line cDNA (10ng), (b) tongue squamous cell carcinoma (TSCC) sample genomic DNA (10ng), and (c) RNA synthesized by IVT in the background of MCF7 cell line RNA (50ng), for performing real-time PCR. For each primer, Ct values (in triplicates) in different concentrations of synthetic DNA/RNA fragments are plotted in the graph. Specificity of the primers to amplify single amplicon is represented as single peak in melting curves generated by “melting curve genotype analysis” in Roche Light cycler 480 machine.

To mimic the complexity of a clinical sample, serial dilutions of the *in vitro* transcribed RNA and viral synthetic DNA (see methods) were used as a template for qRT-PCR/qPCR along with 10ng human cDNA, or 10ng human gDNA, or 50ng human total RNA as background based on SYBR-Green I fluorescence**.** The Ct values for all test primers targeting SARS-CoV-2 *Nucleocapsid* gene (N1 and N2) and *ORF1ab* gene (N3) showed decreasing trend with increasing concentration (1pg, 10pg, 100pg and 1000pg) of the synthetic DNA fragments at a minimal amount of 1pg for both the synthetic DNA and RNA viral templates, while the Ct value for *RNase P* (C1), included as an internal reference control, remained comparable across indicating the constant amount of carrier DNA and RNA. Importantly, the Ct value for no template control wells remained undetermined across all the experiments (Figure 2). The melting curve genotyping analysis for all the primer pairs, except N3 that was excluded from further analysis, showed a single peak suggesting the absence of any non-specific amplification products (Figure 2). Thus, Ct value less than or equal to 30, of products N1, N2, and C1 indicate positive signal for SARS-CoV-2 with the limit of detection at 1pg for synthetic SARS-CoV-2 suggesting high sensitivity of primers to detect small traces of SARS-CoV-2.

**Figure 3:**
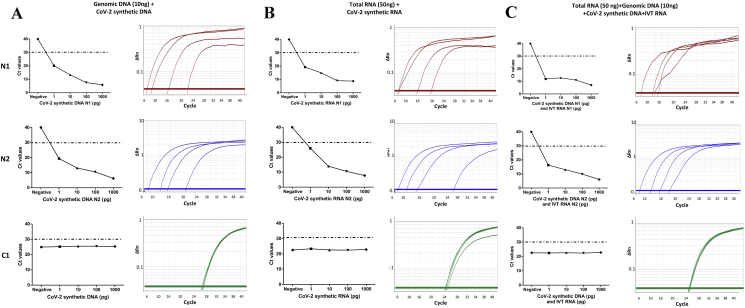
qRT-PCR using TaqMan probe-based detection approach. Real time PCR to determine the sensitivity and specificity of TaqMan probes and primers to detect the SARS-CoV-2 sequence N1, N2 and *RNase P* internal control, C1. For each TaqMan probe, Ct values are plotted against varying concentration of synthetic SARS-CoV-2 DNA and RNA templates in the background of (a) TSCC human genomic DNA (10ng), (b) MCF7 cell line total RNA (50ng) and (c) human MCF7 cell line total RNA and TSCC genomic DNA complexity. Specificity of single amplicon was represented in the respective amplification curve indicating 6-FAM/HEX fluorescence signal emitted by dual labelled probes.

Given that dually fluorophore-labelled hydrolysis TaqMan probes offer an alternative approach to the problem of specificity, we compared the findings obtained using SYBR-Green chemistry by designing TaqMan probes for the detection of the two *Nucleocapsid* gene (N1 and N2) with 6-FAM/BHQ-1 dual labelled fluorophore and quencher, while the control probe targeting the human *RNase P* gene (C1) was tagged with HEX/BHQ-1 dye. Using the *in vitro* synthesized SARS-CoV-2 synthetic N1 and N2 transcripts and DNA fragments serial dilutions of 1pg, 10pg, 100pg and 1000pg were prepared as a template in the background of human total RNA (50ng) or genomic DNA (10 ng) or human total RNA and genomic DNA both. Similar to the findings based on SYBR-Green I dye, the TaqMan qRT-PCR results showed a decrease in Ct value for N1 and N2 with increasing concentration of SARS-CoV-2 *in vitro* transcribed RNA or synthetic DNA or both (Figure 3) that could similarly detect SARS-CoV-2 RNA as low as 1pg in a sample, in the background of human total RNA or human genomic DNA or both suggesting comparable specificity of the N1 and N2 primers and probe to detect SARS-CoV-2 RNA in the sample (Figure 3). The Ct value for C1 remained constant, again indicating a constant amount of carrier DNA or RNA in the reaction. However, consistent with the literature [22], for the same concentration of the N2 synthetic fragments, the corresponding value using TaqMan probes as compared to SYBR Green dye was consistently lower by 3 for Ct value up to 30 cycles.

**Figure 4:**
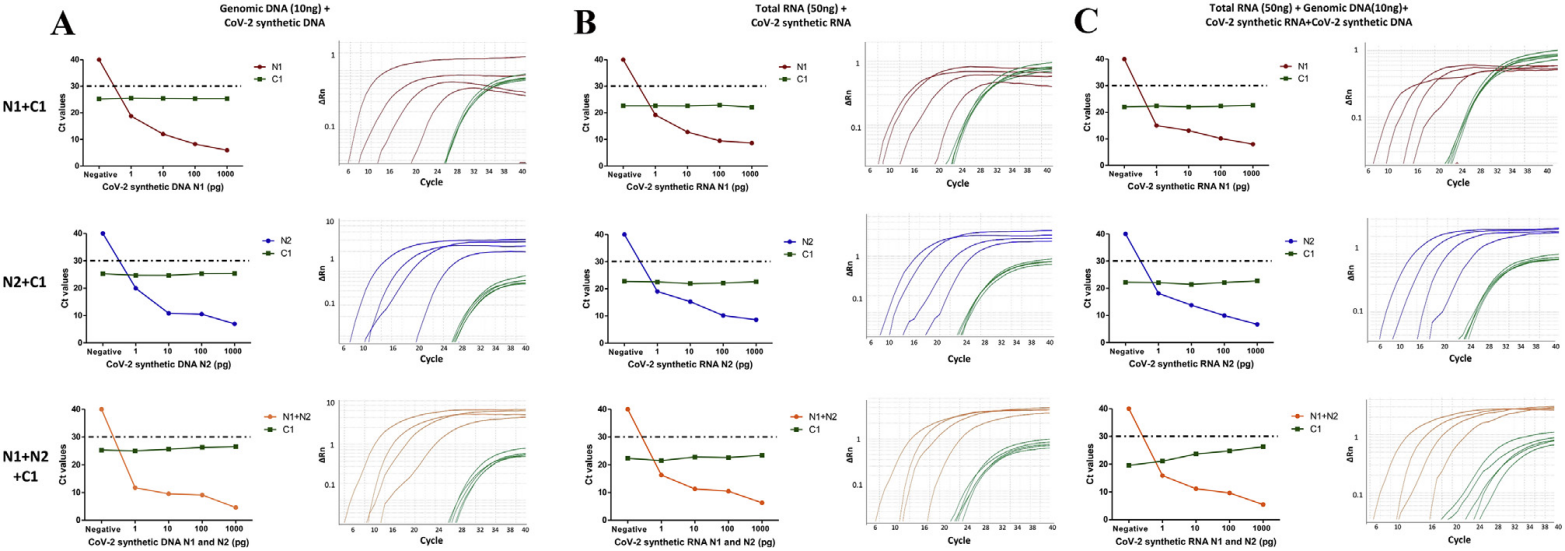
Multiplex TaqMan qRT-PCR to detect SARS-CoV-2. Real time PCR to determine the sensitivity of TaqMan probes to detect the SARS-CoV-2 sequences N1, N2 and C1 in a multiplex qRT-PCR. (a) For each TaqMan probe, Ct values are plotted against varying concentration of synthetic SARS-CoV-2 DNA and RNA templates in the background of (a) TSCC human genomic DNA (10ng), (b) human MCF7 cell line total RNA (50ng) and (c) human MCF7 cell line total RNA and TSCC genomic DNA complexity. Specificity of single amplicon was represented in the respective amplification curve indicating 6-FAM/HEX fluorescence signal emitted by dual labelled probes in multiplex PCR.

With the increasing need for more cost-effective SARS-CoV-2 detection, it is of paramount relevance to detect multiple reference target and internal reference control genes in the same tube, without compromising with the specificity and sensitivity of the viral detection. This is only possible when probe-based multiplexing is performed. Thus, the sensitivity of TaqMan probes for N1, N2 and C1 when combined together in a single reaction, was determined by multiplex qRT-PCR. As described above, serial dilutions for the range of concentrations (1pg, 10pg, 100pg and 1000pg) for SARS-CoV-2 *in vitro* synthesized RNA and synthetic DNA was prepared which was used as a template in the background of human total RNA (50ng) or genomic DNA (10ng) or both. The results of multiplexing qRT-PCR with TaqMan probe N1, N2 and C1 combination showed efficient amplification and detection of both the target regions by their specific primers and probes using synthetic templates. As expected, Ct value for N1 and N2 decrease with increasing concentration of synthetic viral RNA and DNA (Figure 4), without any significant change in the C1 Ct value. Importantly, there was a high concordance between the Ct values for all the test and control probes in multiplex and single reactions PCR with each TaqMan probe, suggesting no decrease in the sensitivity following multiplexing of probe sets.

### 3.3 COVID qPCR analyzer, an automated tool to quantitatively analyze and interpret the qRT-PCR assay

To minimize variability and automate the qRT-PCR downstream analysis of multiple samples in the same run, we further developed a GUI based automated *in silico* analytical tool, COVID qPCR Analyzer (www.actrec.gov.in/pi-webpages/AmitDutt/Covid/Analyzer.html), to detect the virus in an unambiguous and reproducible manner. The tool poses a minimal requirement of third-party tools and is compatible with output obtained from any real-time PCR machine -- in a platform-independent manner, ensuring its universal application and adaptability across laboratories. The COVID qPCR Analyzer allows the flexibility to adjust the threshold of Ct value to fall within the exponential phase of the fluorescence curves and above any background signal based on the reference sample. A positive result is indicated with a Ct value of less than the defined threshold with simultaneous detection for two regions of SARS-CoV-2 *Nucleocapsid* gene, irrespective of the Ct value determined for the internal reference control gene *RNase P*. A negative result is indicated with a Ct value of higher than the threshold for the two regions of SARS-CoV-2 *Nucleocapsid* gene, with a Ct value below the threshold for the internal reference control gene *RNase P*. In case the Ct value of the SARS-CoV-2 *Nucleocapsid* gene and the internal reference control gene *RNase P* exceeds the threshold or remains undetermined, indicate an inconclusive outcome with a requirement to repeat the test (Table 1).

**Table 1:** Metric for data analysis and interpretation.

N1+N2	C1	Result
+	+	SARS-CoV-2 detected
+	−	SARS-CoV-2 detected
−	+	SARS-CoV-2 not detected
−	−	Inconclusive result

+	Ct value equal to or less than 30
−	Ct value more than 30

### 3.4 Clinical validation and accuracy of the one-step, one tube, multiplex probe-based qRT-PCR assay

A total of 26 clinical samples were investigated with two primer sets for SARS-CoV-2 *Nucleocapsid* gene (N1 and N2) and a primer set for human *RNase P* gene (C1) by real-time RT-PCR. The data was analysed using COVID qPCR Analyzer, wherein a positive result was indicated with a Ct value equal to or less than 30; negative result with a Ct value of higher than 30 for the two regions of SARS-CoV-2 *Nucleocapsid* gene and internal reference control gene *RNase P*. 5 of 26 patient samples were confirmed to be infected with SARS-CoV-2, while 21 tested negative (Table 2), consistent with the gold standard outcomes obtained using commercial kits. Thus, the sensitivity was 100% and specificity was 100%. A total of 115 validations were performed for SARS-CoV-2 *Nucleocapsid* genes and 69 validations for the human *RNase P* gene (C1) by real-time RT-PCR with several dilutions ranging from 1-100 ng of the clinical samples. In addition, the accuracy of the one-step, one-tube, a multiplex probe-based real-time PCR assay was determined with the *in vitro* synthesized SARS-CoV-2 RNA synthetic transcripts in the background of human total RNA. The real-time RT-PCR reactions performed detected Ct value with the limiting amount of 500pg of the total template with SYBR Green I dye and 50pg with the TaqMan probe-based methodology, below which Ct values couldn't be determined. Using the graphical interface of the NEBiocalculator, the limit of detection (LoD) was determined to be 150 and 15 RNA copies/reaction for the SYBR Green dye-based and TaqMan probe-based methodologies (https://nebiocalculator.neb.com/#!/ssrnaamt).

**Table 2:** Clinical validation of the one-step, one-tube, multiplex probe-based real-time PCR assay.

No. of samples	Gold standard		One-step, one-tube, multiplex probe-based real-time PCR assay		Sensitivity (%)	Specificity (%)	Accuracy (%)
	Virus positive	Virus Negative	Virus positive	Virus negative			
26	5	21	5	21	100	100	100

## 4 Discussion

We describe a comparative account of qRT-PCR based detection of SARS-CoV-2 using SYBR Green I and Taqman probes. Although SYBR Green-based qRT-PCR protocol is comparatively economical, the non-specific binding of the dye to DNA resulted in low specificity and sensitivity with limit of detectability of 150 copies of viral RNA molecules/reaction, whereas TaqMan probe-based protocol could detect as low as 15 copies of viral RNA molecules/reaction suggesting higher sensitivity. Moreover, consistent with the guidelines by WHO and for the reliability of results wherein at least two genomic targets are required for diagnostic test [8], the TaqMan probe based assay allows to multiplex the two viral specific target probes along with the human control probe, labelled by different fluorophores, in the same tube as a one-step, one-tube multiplex probe-based qRT-PCR reaction.

Furthermore, in comparison to 13 different SARS-CoV-2 detection kits (6 based on qRT-PCR; 5 based on RT-LAMP; and 2 based on CRISPR-Cas12-RT-LAMP) reported in the literature (Supplementary Table S4) [4, 5, 13, 14, 23, 24, 25, 26, 27, 28, 29, 30, 31], the one-step, one-tube, multiplex probe-based real-time PCR assay derives its strength from the fact that it can be offered at the point-of-care testing for the detection of SARS-CoV-2 by using dual TaqMan test probes, both labelled with 6-FAM/BHQ-1, targeting unique regions of the SARS-CoV-2 *Nucleocapsid* gene along with an internal reference control gene *RNase P* probe labeled with HEX-1/BHQ-1, simultaneously. Along with the RNA extraction process, the qRT-PCR and analysis can be completed in less than 2h that can be easily compiled and delivered in a form of an automated report generated using COVID qPCR Analyzer, a GUI based *in silico* analytical tool, in an unambiguous and reproducible manner, making it highly cost-effective. Moreover, the GUI allows the user to set an adjustable threshold Ct value based on the reference samples correcting for the individual bias that may vary between runs due to varying template quality and ensures the universal applicability of the automated analyzer tool, based on single target probe with subsequent provisions for customization to adapt for multiple target test probes.

A major shortcoming of the one-step, one-tube, multiplex probe-based real-time PCR assay is that the primer sequences designed for the two regions within the SARS-CoV-2 *Nucleocapsid* gene is based on the alignment of in 93 SARS-CoV-2 genomes isolates compared against 27,399 human-specific SARS/SARS-like genomes obtained from NCBI-virus database [15]. However, with the pandemic status of COVID-19, the accuracy of the one-step, one-tube, multiplex probe-based real-time PCR assay may be affected by the variations arising within the primers to amplify the target genes and affecting the binding of the TaqMan probes designed within the target regions. A complementary approach with the availability of whole genome sequencing of the evolving SARS-CoV-2 sequence would help to address the variability and update the assay described here. Secondly, the real time-RT PCR assay involves intensive laboratory steps with a requirement of skilled manpower to perform the assay that requires a modern molecular laboratory infrastructure and an expensive instrument. Thirdly, the accuracy of the one-step, one-tube, multiplex probe-based real-time PCR assay needs to be further verified and evaluated using a large number of clinical samples. Thus, there remains an unmet need to simplify the detection at the point of care in a portable manner. Lastly, as a general shortcoming of the real time-RT PCR based negative outcome of SARS-CoV-2 test does not necessarily rule out infection in the course of an individual patient's disease.

In conclusion, the one-step, one-tube, multiplex probe-based real-time PCR assay with automated qPCR analyzer described in this study demonstrates high diagnostic sensitivity and specificity using clinical samples to detect the presence of SARS-CoV-2. Given the lack of effective therapeutic intervention available such as vaccines or treatments, early diagnosis of COVID-19 is the most crucial alternative to reduce SARS-CoV-2 transmission by identifying and isolating infected individual to flatten the spread of the COVID-19 virus. The assay may be further evaluated across multicentre as prospective studies in the context of clinical management during the prevailing COVID-19 outbreak and may be of high significance for different laboratories to set a diagnostic facility in an economical way. An effective and validated one-step, one-tube, multiplex probe-based real-time PCR assay described can be easily adapted by the community and would help further improve our understanding of the current estimates of COVID-19 case-fatality rates that are most likely inflated in the current scenario due to the preferential availability of data from countries with better diagnostic capabilities, underscoring the significance of universal availability of accurate diagnostic testing methodologies during the COVID-19 pandemic.

## Declarations

### Author contribution statement

B. Dharavath and N. Yadav: Conceived and designed the experiments; Performed the experiments; Analyzed and interpreted the data; Contributed reagents, materials, analysis tools or data; Wrote the paper.

S. Desai and A. Dutt: Conceived and designed the experiments; Analyzed and interpreted the data; Contributed reagents, materials, analysis tools or data; Wrote the paper.

R Sunder, P. Bhanshe, A. Gupta and A. Redhu: Performed the experiments; Analyzed and interpreted the data.

R. Mishra, N. Patkar, S. Dutt and S. Gupta: Analyzed and interpreted the data; Contributed reagents, materials, analysis tools or data.

M. Ketkar: Analyzed and interpreted the data; Wrote the paper.

### Funding statement

A. Dutt was supported by 10.13039/501100010489Advanced Centre for Treatment, Research and Education in Cancer.

### Competing interest statement

The authors declare no conflict of interest.

### Additional information

No additional information is available for this paper.

## Appendix A Supplementary data

Supplementary File1 Supplementary File1

Supplementary_FigureS1 Supplementary_FigureS1
