# Supplementary material for: A one-step, one-tube real-time RT-PCR based assay with an automated analysis for detection of SARS-CoV-2
Source: Heliyon. 2020 Jul 7;6(7):e04405. doi: 10.1016/j.heliyon.2020.e04405 (PMC7341355; doi:10.1016/j.heliyon.2020.e04405)
Supplement: Supplementary File1 [file mmc1.pdf]

TATA MEMORIAL CENTRE
ADVANCED CENTRE FOR TREATMENT RESEARCH & EDUCATION IN CANCER

A One-step, One-tube Real-time PCR Based Assay with an Automated Analysis for Detection of SARS-CoV-2 (Manual)

1. RNA extraction protocol from buccal swab
2. A One-step, One-tube Real-time PCR Based Assay
3. SARS-CoV-2 RT-PCR Analyzer

Bhasker Dharavath, Neelima Yadav, Sanket Desai, Roma Sunder, Rohit Mishra, Madhura Ketkar, Prasanna Bhanshe, Anurodh Gupta, Archana Kumari Redhu, Nikhil Patkar, Shilpee Dutt, Sudeep Gupta, Amit Dutt

RNA extraction protocol from buccal swab

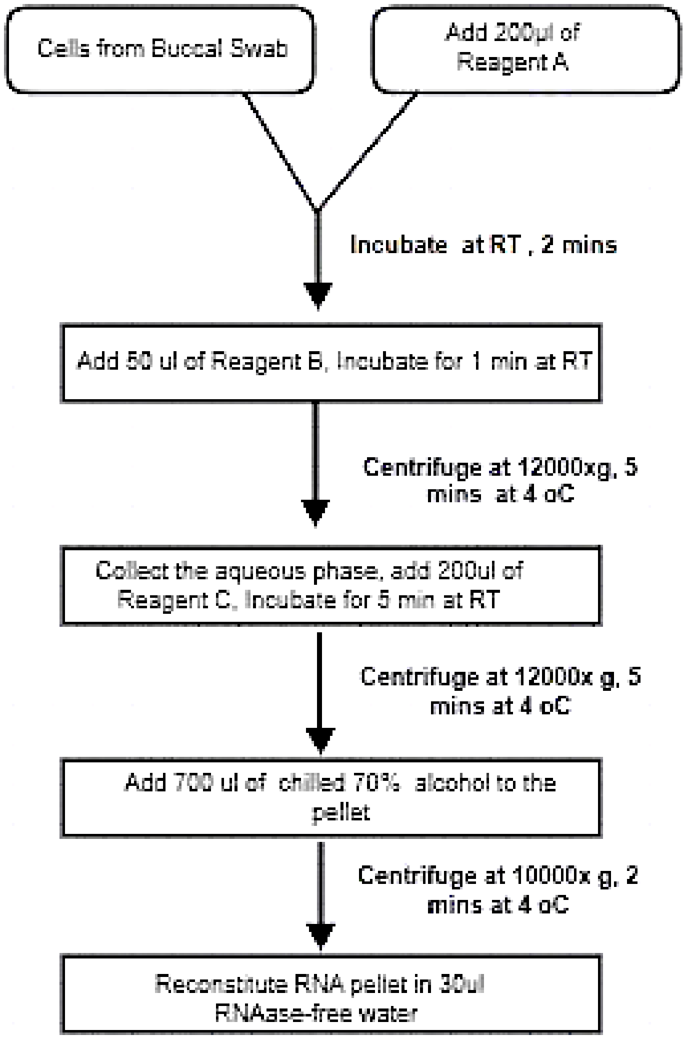

1. The protocol is standardised for an easy, quick and convenient method to isolate total RNA from buccal swab. The isolated total RNA with following extraction protocol can be used directly to perform qRT-PCR for detection of SARS-Cov-2 sequences.
2. To extract the cells from the inside of patient's cheeks using blunt end of the sterile wooden toothpick, insert the toothpick and rub the inside of the cheeks for at least 1 minute. Ensure that the patient has not eaten or drank anything 30 minutes prior to this procedure.
3. Place this toothpick into a tube containing 200ul PBS. To make sure that the sample material is well resuspended in PBS, vigorously swirl the swab in the tube.

4. Centrifuge the tube at 12000 x g for 2 minutes at room temperature (RT). Discard the supernatant and add 200 ul of reagent A to the pellet. Gently resuspend the pellet in reagent A with the help of a pipette. Let the lysis occur by incubating the tube at RT for 2 minutes
5. Add 50 ul of reagent B to the tube and mix the solutions well by shaking the tube in up-down motion for 15-20 seconds. Let the tube rest for 1 minute at RT and centrifuge at 12000 x g for 5 min at 4°C.
6. After centrifugation, three distinct layers should be formed i.e (i) uppermost colourless aqueous phase (RNA) (ii) white-coloured interphase (DNA) (iii) lowest pink-coloured phase (proteins and other debris); from which collect the uppermost layer meticulously in a new tube. Make sure not to touch the DNA layer with the tip as even a minute amount of DNA can contaminate the RNA extraction.
7. Add 200 ul of reagent C to this tube containing only aqueous phase and mix the contents by inverting the tube. Let the tube stand at RT for 5 minutes.
8. Centrifuge the tube at 12000 x g for 5 minutes at 4°C. Discard the supernatant and wash the pellet by gently adding 700 ul of chilled 70% ethanol to the tube without resuspending the pellet with ethanol.
9. Immediately centrifuge at 10000 x g for 2 minutes at 4°C, discard the supernatant and air dry the pellet.
10. Reconstitute this pellet in 30ul of RNase-free water. Conduct quantitative analysis to determine its concentration.
11. Place RNA in ice for immediate downstream process or store at -80°C.

- **Reagents composition:**

**Reagents**	**Composition**
Reagent A	TRI Reagent R (Sigma)
Reagent B	Choloroform
Reagent C	Isopropanol

- **Approximate time for the RNA extraction :** 21 minutes

A One-step, One-tube Real-time PCR Based Assay

Set up of reaction:

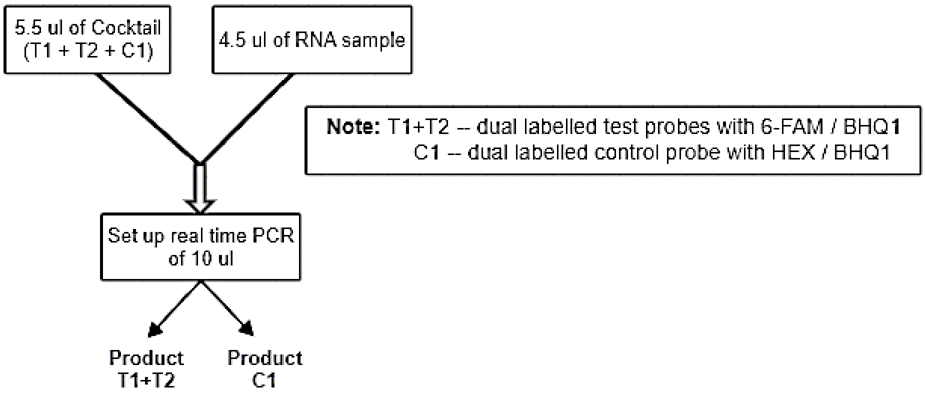

Note: T1+T2 -- dual labelled test probes with 6-FAM / BHQ1
C1 -- dual labelled control probe with HEX / BHQ1

Temperature condition for multiplex real time PCR

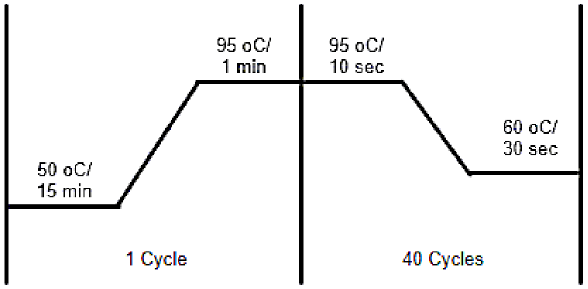

In the multiplex real-time PCR set up, select following dye and quencher options
For T1 and T2: dye = FAM; quencher = BHQ1
For C1: dye = HEX; quencher = BHQ1

Threshold cut-off : Ct value less than 30 to consider as a positive signal

Data analysis and Interpretation

T1+T2	C1	Comments
+	+	SARS-CoV-2 detected
+	−	
−	+	SARS-CoV-2 not detected
−	−	Inconclusive results

Instructions

A. Setting up the reaction with cocktail:

B. Take 4.5 ul of total RNA sample (minimum 20 ng/ul)/ positive control RNA/ negative control RNA and add in 5.5 ul of kit cocktail. Tap the mixture properly and incubate in real time PCR machine for the following temperature conditions:

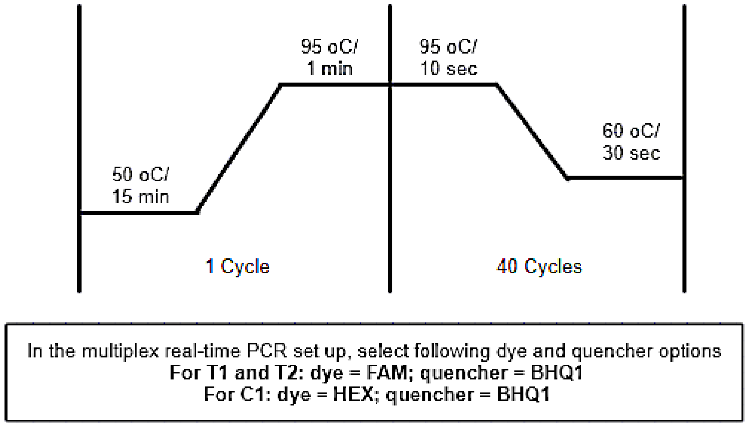

Figure 1. Thermocycler temperature condition for Reaction

C. While setting the real time PCR program for the reaction, select 6-FAM and HEX fluorescent reporter dye for T1+T2 and C1 respectively in multiplex PCR setting. Also, both the probes have BHQ-1 quencher. Thereby, set either BHQ-1/None in the program for quencher selection.

Important note:

D. The provided temperature conditions would be able to synthesize the cDNA from human total RNA sample and further amplify the target regions. For synthesis and amplification of SARS-CoV-2 targeted sequences, two pairs of primers and TaqMan probes (T1 and T2) are provided in the cocktail mixture. Additionally, one more pair of primers and TaqMan probe (C1) is included in the cocktail for detection of housekeeping control gene (RNase P).

E. For discrimination between the signal from T1+T2 or C1 in multiplex qPCR, both the TaqMan probes are labelled with different fluorescence dye with different emission wavelengths. Fluorescent dye tag for TaqMan probes T1+T2 and C1 are 6-FAM (maximum emission wavelength, 518 nm) and HEX (maximum emission wavelength, 556

nm) respectively. In some real time PCR machines (for example- AB1, Quant Studio 5) if options for HEX/6-FAM are unavailable, then choose fluorescent dye with nearby emission wavelength. For example: VIC (max. emission wavelength, 554 nm) can be selected for HEX dye. Also, make sure to select the “ROX” dye as a passive reference dye.

a. For more number of RNA samples, For example – for 70 RNA samples, same reaction can be set up in 96 well PCR plate (plate not provided in kit) as follows: each well represent RNA sample from different individual (B1, B2, B3…RNA sample from individual 1, individual 2, individual 3 in cocktail and so on).

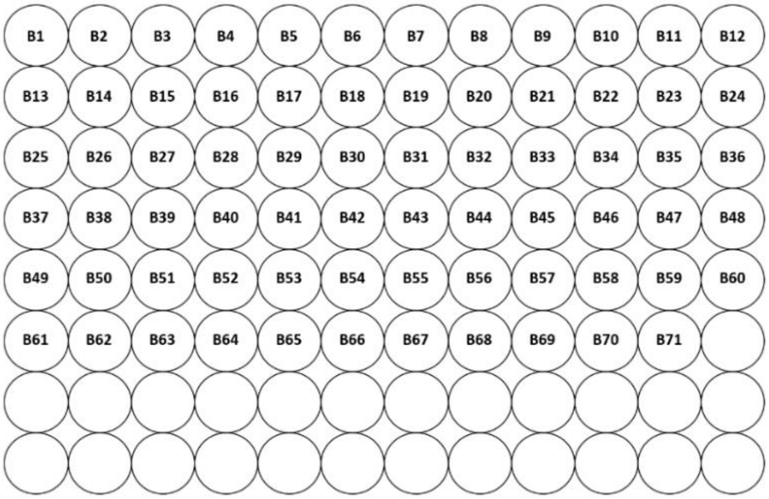

Figure 2. Schematic of 96- well PCR plate for reaction with different RNA samples.

Also, it is suggested to include following control reactions in the test plate:

a. **“No template control”** for kit cocktail.
b. **“Negative template control”** using kit cocktail and negative RNA sample provided with the kit.
c. **“Positive template control”** using kit cocktail and positive RNA sample provided with the kit.

1. Data Analysis and Interpretation :

- The threshold cut off Ct value for T1+T2 and C1 products will be less than 30. Less than 30 Ct value for T1+T2 indicates positive signal for SARS-CoV-2.
- For positive control RNA (provided with kit), the expected Ct values are:
  1) Ct value for T1+T2 = 14-16

2) Ct value for C1 = 22-24

- For negative control RNA (provided with kit), the expected Ct values are:

  1) Ct value for T1+T2 = Undetermined

  2) Ct value for C1 = 22-24

- Following is the summary table based on which comments can be drawn for positivity/negativity and invalidity of the experiment for each RNA sample.

Data analysis and Interpretation

T1+T2	C1	Comments
+	+	SARS-CoV-2 detected
+	−	
−	+	SARS-CoV-2 not detected
−	−	Inconclusive results

Table. Summary of interpretation of data in case of different outcomes.

Additional Information

- Following is the compositions of cocktails in the ACTREC-SARS-CoV-2 detection kit:

**Composition of cocktail**		
**Reagent**	**For 1 reaction**	**For 5 reactions**
KiCqStart One-Step Probe RT-qPCR ReadyMix (Low ROX)	5 ul	25 ul
Forward primer_T1	0.025 ul	0.13 ul
Reverse primer_T1	0.025 ul	0.13 ul
Forward primer_T2	0.025 ul	0.13 ul
Reverse primer_T2	0.025 ul	0.13 ul
Forward primer_C1	0.025 ul	0.13 ul
Reverse primer_C1	0.025 ul	0.13 ul
TaqMan probe_T1	0.02 ul	0.1 ul
TaqMan probe_T2	0.02 ul	0.1 ul
TaqMan probe_C1	0.02 ul	0.1 ul
ROX dye (Low)	0.2 ul	1 ul
**Total volume**		**27 ul**

- Approximate time for the experimental steps:

Total reaction time	71 min

- Reagents provided in the kit are for 200 RNA samples.

**Reagent provided**	**Volume provided**
Cocktail	27 ul
Positive control RNA sample	20 ul
Negative control RNA sample	20 ul

- Fluorescent and quencher details of TaqMan probes:

**TaqMan Probe**	**Fluorescent dye**	**Quencher**	**Absorption wavelength (nm)**	**Emission wavelength (nm)**
T1+T2	6-FAM	BHQ-1	494	517
C1	HEX	BHQ-1	535	556

The COVID qPCR Analyzer

Pre-requisites required for installation of Covid qPCR Analyzer

The GUI based automated tool is developed in R programming environment. As a prerequisite, it depends of the R programming language and certain packages. Please follow the below instructions to set up your machine for the Covid qPCR Analyzer.

1. Installation instructions for R (in Windows and Linux OS)

Suggested version of R is Version > 3.5

To Install R in Windows:
- Open an internet browser and go to www.r-project.org.
- Click the "download R" link in the middle of the page under "Getting Started."
- Select a CRAN location (a mirror site) and click the corresponding link.
- Click on the "Download R for Windows" link at the top of the page.
- Click on the "install R for the first time" link at the top of the page.
- Click "Download R for Windows" and save the executable file somewhere on your computer.
- Run the .exe file and follow the installation instructions.

To Install R in Linux (on Ubuntu OS):
Update the system package and upgrade all our installed packages using the following commands in Terminal

```
$ sudo apt update
$ sudo apt -y upgrade
$ sudo apt -y install r-base
```

2. Installation of packages required for running the tool

Following R packages (available on the CRAN repository https://cran.r-project.org/) are required to be installed for usage of the tool:

- gWidgets2
- digest
- dplyr
- tidyr
- readxl

3. Obtaining Covid qPCR Analyzer tool:

COVID qPCR Analyzer can be downloaded from www.actrec.gov.in/pi-webpages/AmitDutt/Covid/Analyzer.html. The download link on the page will take user to a google form which needs to be submitted to obtain a download link. User is required to click on the download link to obtain the zipped package (CovidqPCRAnalyzer.zip). Upon download, extract the zip folder. Inside the resulting directory, the start program is present inside “src” directory. There is no need to run any installer for this program, the program can be directly executed as below.

<u>Execution in Linux</u>

```
$ unzip COVIDqPCRAnalyzer.zip
$ cd COVIDqPCRAnalyzer
$ Rscript src/COVIDqPCRAnalyzer.R
```

<u>Execution in Windows</u>

1. Extract COVIDqPCRAnalyzer.zip
2. open CMD
3. Enter:
   "<PATH to Rscript program>" "<Path to R file COVIDqPCRAnalyzer.R>"
   e.g.
   "C:\Program Files\R\R-*.*.*\bin\Rscript.exe" "C:\Users\Desktop\COVIDqPCRAnalyzer\src\COVIDqPCRAnalyzer.R"

Guide to use GUI of Covid qPCR Analyzer

COVID qPCR Analyzer graphical user interface is developed for the analysis of real time PCR data to detect the infection of SARS-CoV-2 in a quick, efficient and robust manner.

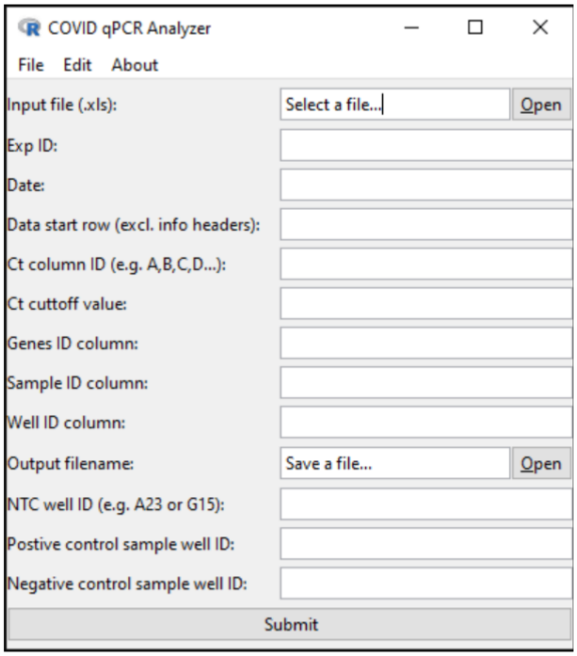

Graphical user interface of COVID qPCR Analyzer

COVID qPCR Analyzer usage instructions:

COVID qPCR analyser provides the function to select the various variables of qRT-PCR result file. Following are the fields that are required to be filled before proceeding the run:

1. **Input file (.xls)**
   Select the real time PCR result file (containing Ct values for tested samples) in this field. The file format accepted by the tool is excel (.xls).

2. **Exp ID**
   This field requires the unique run ID/name of the experiment that user will be using in the analysis.

3. **Date**
   Date of experiment run is required to be filled in this field.

4. **Data start row (excl. info headers)**
   Fill the field with number of row from where the actual Ct value data starts in the qPCR result file. Fill the row number after excluding the header rows

which usually contain the information of real time PCR machine and other technical details.

5. **Ct Column ID (a,b,c,d)**
   Fill the field with the column ID (e.g. A or B or C or D) which contains the Ct values of target and control genes.

6. **Ct cutoff value**
   Fill the field with the cutoff Ct value of real time PCR result. This threshold value is required by the tool for analysing the results -"positive", "negative" or "invalid".

7. **Gene ID column**
   Fill the field with the ID of the column (e.g. A or C or H) with Target gene and control gene ID information in real time PCR raw data file.

8. **Sample ID column**
   Fill the field with the ID of the column (e.g. A or C or H) with Patient sample ID information in real time PCR raw data file.

9. **Well ID column**
   Fill the field with the ID of column (e.g. A or C or H) with Well position information in real time PCR raw data file.

10. **Output filename**
    Choose the destination folder location and output filename.

11. **NTC well ID (e.g. A23 or G15)**
    Fill the field with the Well ID (with column ID and row number, for ex: A23 or G15) of the real time PCR plate, in which reaction with no template control is loaded.

12. **Positive control sample well ID**
    Fill the field with the Well ID (with column ID and row number, for e.g.: A23 or G15) of the real time PCR plate, in which reaction with Positive control sample is loaded.

13. **Negative control sample well ID**

Fill the field with the Well ID (with column ID and row number, for e.g.: A23 or G15) of the real time PCR plate, in which reaction with Negative control sample is loaded.

Once all the information is filled in GUI, proceed with the run by clicking at **“Submit”** button. As soon as the user starts the run, tool will generate the output file which contain the results of SARS-CoV-2 infection presence/absence. The run time for COVID qPCR would be fractions of seconds. Thereby, make this quick tool for real time PCR data analysis.

COVID qPCR Analyzer report:

The output report generated upon running the analyzer is in a .CSV file format and contains the following five columns:

1. **Sample ID name**
   This column will have all the samples names which has been analysed by COVID qPCR analyzer using GUI.

2. **Well ID column**
   This column contains the information of wells ID tested for the analysis.

3. **Exp. ID**
   This column will have the same experiment ID that was given by user while submitting the details in GUI.

4. **Test result**
   This column will contain the results generated by COVID qPCR analyzer. For example-based on specified Ct value the samples would be called “positive”, “negative” and “invalid” for SARS-CoV-2 infection.

5. **Date**
   This column will give the date on which the experiment run was conducted.
