# Supplementary material for: A one-step, one-tube real-time RT-PCR based assay with an automated analysis for detection of SARS-CoV-2
Source: Heliyon. 2020 Jul 7;6(7):e04405. doi: 10.1016/j.heliyon.2020.e04405 (PMC7341355; doi:10.1016/j.heliyon.2020.e04405)
Supplement: Supplementary_FigureS1 [file mmc2.docx]

**Supplementary Figure S1:**


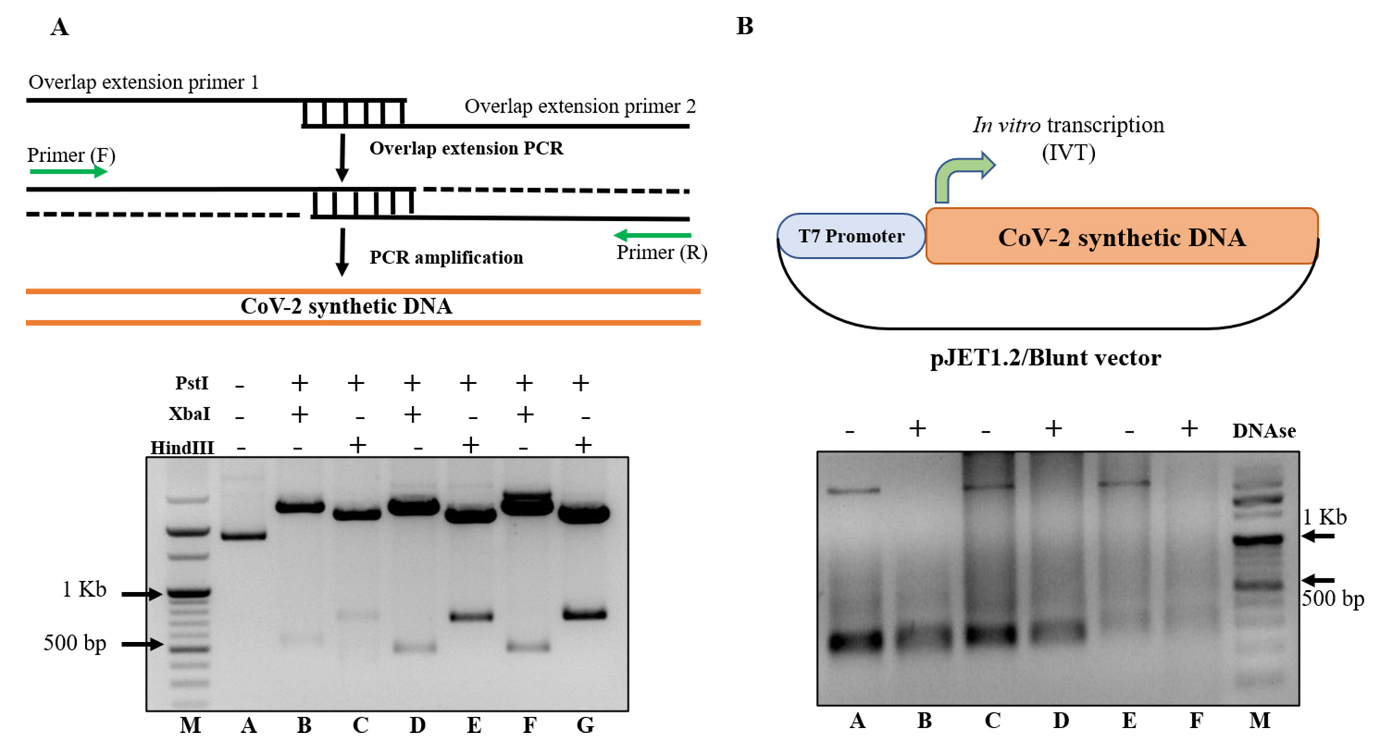


**Supplementary Figure 1.** Generation of SARS-CoV-2 positive template control DNA and RNA

(a) Schematic depiction to illustrate generation of double stranded SARS-CoV-2 synthetic DNA fragments by overlap extension PCR method. Three SARS-CoV-2 positive template controls (N1, N2 and N3) are cloned in pJET1.2/blunt vector and confirmed by restriction digestion with combination of restriction enzymes by agarose gel electrophoresis. Lane M contain 100 bp DNA ladder. Lane A-G consists of restriction digested plasmids with multiple enzyme pairs (A-Undigested pJET/1.2 plasmid; B, D and F – XbaI and PstI digested cloned constructs of N1, N2 and N3 respectively; C, E and G - PstI and HindIII digested cloned constructs of N1, N2 and N3. (b) Schematic depiction illustrating in vitro transcription for synthesis of SARS-CoV-2 RNA fragments from N1, N2 and N3 fragments cloned in pJET/1.2 vector. Agarose gel electrophoresis results confirmed the synthesis of RNA by in vitro synthesis. Lane M contain 100 bp DNA ladder. In the lanes followed by DNA ladder, DNase treated (+) and untreated (-) IVT RNAs of the three fragments are loaded in the order N2, N3 and N1 from left.
